# Rheological, Thermal, and Degradation Properties of PLA/PPG Blends

**DOI:** 10.3390/ma12213519

**Published:** 2019-10-26

**Authors:** Dong Xie, Yang Zhao, Yuan Li, Anna Marie LaChance, Jinqing Lai, Luyi Sun, Junjia Chen

**Affiliations:** 1 Guangdong Provincial Bioengineering Institute (Guangzhou Sugarcane Industry Research Institute), Guangzhou 510316, China; xd0929@163.com (D.X.); drzhaoy88@163.com (Y.Z.); sichuanliyuan@sina.com (Y.L.); 2 Guangdong Biomaterials Engineering Technology Research Center, Guangzhou 510316, China; 3 Polymer Program, Institute of Materials Science and Department of Chemical & Biomolecular Engineering, University of Connecticut, Storrs, CT 06269, USA; anna.marie@uconn.edu

**Keywords:** polylactic acid, polypropylene glycol, rheology, degradation, plasticization

## Abstract

The work presented herein focuses on simulating the compounding process via a torque rheometer, as well as the relationship between the melt viscosity and the polymer molecular weight (MW). We aim to predict the plasticization of polylactic acid (PLA) using polypropylene glycol (PPG) with different MWs. The rheological properties of the PLA/PPG composites containing PPG with different MWs were systematically studied by capillary rheometry and torque rheometry. The initial degradation of PLA/PPG composites during melt processing was monitored in real time. The results indicate that PPG can significantly reduce the melt viscosity of PLA/PPG composites, leading to obvious pseudoplastic fluid behavior. The lower the MW of PPG, the lower the viscosity of the PLA/PPG composite. The addition of PPG was favorable for the degradation of PLA during processing, and the degradation degree of the composite materials increased as the MW of PPG was decreased.

## 1. Introduction

Nowadays, plastic products are widely used across the world, which brings great convenience to human life. As estimated, around 8.3 billion tons (Bt) of plastics were produced from 1950 to 2015, among which 9% was recycled, 12% burned, and 79% accumulated in landfills or nature [1]. However, most plastics need hundreds of years to be completely degraded in nature. Such serious environmental pollutions brought by non-degradable plastics ultimately endanger our health once released into nature and thus should never be neglected [2,3,4,5].

Poly (lactic acid) (PLA) [6,7,8] is a typical aliphatic polyester, which is made by a microbial fermentation product, lactic acid. Being non-toxic, completely biodegradable, highly transparent, strong, and stiff, PLA is one of the ideal materials to replace traditional plastics to alleviate environmental issues. However, there are still several limitations for utilizing PLA as an ideal sustainable polymer material with respect to its mechanical properties. PLA is a rigid and brittle polymer at room temperature. The high Young’s modulus and low elongation at break of PLA limit its applications in areas requiring high toughness and stretchability [9]. The plasticization modification of PLA by citrate acid ester, epoxy soybean oil (ESO), polyethylene glycol (PEG), etc., can effectively improve the brittleness of PLA [10,11,12,13,14]. Polypropylene glycol (PPG) and PEG, with a similar polyether structure, possess a good plasticizing effect on PLA. Piorkowska et al. [15,16] compared the plasticizing effect of PPG on PLA with two molecular weights (MWs) of 425 and 1000 g/mol. Their result indicated that PPG modified the mechanical properties of the resultant composites by altering the crystallinity of PLA. PPGs are viscous liquids in the MW range of 150–4000 g/mol. They do not crystallize and have a low glass transition temperature ranging from −60 to −75 °C [17]. Ke et al. [18] found that PPG and PLA had poor compatibility, showing inert dilution. This phenomenon can reduce the initial crystallinity of PLA. Lezak et al. [19] plasticized PLA/flax fiber composite by PPG, which reduced the cold crystallization temperature of PLA and improved the crystallinity of the composite.

Among the existing work of PPG plasticized PLA, researchers paid more attention to the mechanical and crystallization properties of the plasticized PLA. However, investigations on the degradation of PLA during processing as a function of PPG MW and content have been rarely reported. A related study from Signori et al. reported that biodegradable thermoplastics were particularly susceptible to the thermal and thermal oxidative degradation during processing, as evidenced by a reduced MW and deteriorated properties after processing [20,21,22,23,24,25]. Meanwhile, Costa et al. proved that melt viscosity was very sensitive to slight changes in MW, whereas the torque of the polymer depends on its melt viscosity [26]. In this case, the torque decreases along with the degradation of polymer during processing. Therefore, characterization of the torque of a polymer by rheological methods can be used to study the changes in MW because of the degradation. Based on this principle, the degradation rate of materials under certain processing conditions can be further evaluated [27,28,29,30].

The work presented herein focuses on the modification of PLA’s brittleness and degradation. During processing, PLA was plasticized by PPG with different MWs. And the effect of the PPG MW on the melt fluidity of the mixed system was investigated by capillary rheometer. According to the sensitivity of the melt viscosity to the MW of the polymer, the relationship between the torque change and the MW drop was established by the data characterized by a torque rheometer [27]. The initial degradation of the PLA/PPG composites during melt processing was estimated in real time. At the same time, the thermal properties and the micro-morphology of the PLA/PPG composites plasticized by different MWs of PPG were systematically investigated.

## 2. Experimental

### 2.1. Materials

PLA (Ingeo TM Biopolymer, 2003D) was supplied by NatureWorks LLC (Minnetonka, MN), with a density of 1.24 g/cm^3^ and a weight average MW (*M_w_*) of 112,000 g/mol. The PLA resin was dried in an oven at 80 °C for 10 h before use. PPG was supplied by Guangzhou Runhong Chemical Co., Ltd. (Guangzhou, China), with MWs of 200, 400, 600, 800, and 1000 g/mol.

### 2.2. Preparation of PLA/PPG Composites

The PLA/PPG composites were prepared by adding 5 phr PPG with various MWs (200, 400, 600, 800, and 1000 g/mol). PLA and PPG were dried in an oven at 80 °C for 10 h, melt blended at 170 °C with a torque rheometer (Rheo Drive 7, Thermo Fisher Scientific, Karlsruhe, Germany), and then pulverized. The pellets of the PLA/PPG composites were dried in an oven at 80 °C for 10 h before the capillary rheological and thermal characterizations. The composites were then injection molded into standard specimens for thermal and micro-morphological characterizations.

### 2.3. Characterization

The flow properties of the PLA/PPG composites were investigated by a capillary rheometer (Rheologic 5000, Ceast, Torino, Italy) at 170 °C with a shear rate of 50–3000 s^−1^. The melt flow properties of the PLA/PPG composites during processing were investigated by a torque rheometer (Rheo Drive 7, Thermo Fisher Scientific, Karlsruhe, Germany). The trend of the melt torque of the PLA/PPG composites as a function of processing time and temperature was investigated. The thermal properties of the PLA/PPG composites were characterized by a differential scanning calorimetry (DSC Q20, TA Instruments, New Castle, DE, USA) under a nitrogen atmosphere. During the measurement, the temperature was raised from room temperature to 200 °C at a heating rate of 10 °C /min, and then cooled to 0 °C at a rate of 10 °C/min. Then, the temperature was raised to 200 °C again at a heating rate of 10 °C/min, and the thermogram of the second heating process was recorded. The thermal stability of the PLA/PPG composites was investigated using a synchronous thermal analyzer (Jupiter STA 449F3, Netzsch, Selb, Germany) under a nitrogen atmosphere. Herein, the temperature was raised from room temperature to 600 °C at a heating rate of 20 °C/min. Scanning electron microscopy (FEI Phenom Prox, operated at 5 kV, Phenom-world B.V., Eindhoven, Netherlands) was used to observe the micro-morphology of the liquid nitrogen fractured surface of the PLA/PPG composites. The fracture surface was sputter coated with gold prior to imaging. 

### 2.4. Methodology

#### 2.4.1. Determination of the Polymer Non-Newtonian Index

Polymers are viscoelastic and can be oriented by a shear force in a viscous flow state so polymer melts have non-Newtonian fluid properties [31]. For non-Newtonian fluids, the relationship between shear stress, shear rate, and flow index *n* (non-Newtonian index) is expressed by Equations (1) and (2) [32], where shear stress *σ* can be obtained from apparent viscosity $\eta_{a}$ and shear rate $\dot{\gamma }$; *K* is the apparent viscosity index; *n* is the non-Newtonian index, which is commonly used to describe the fluidity of a polymer in a viscous flow state.

(1) $$\sigma =K{\dot{\gamma }}^{n}=\eta_{a}\dot{\gamma }$$

(2) $$n=\frac{d\ln\sigma_{a}}{d\ln{\dot{\gamma }}_{a}}$$

#### 2.4.2. Determination of Polymer Degradation Rate by TORQUE Rheometry

By using a torque rheometer, the torque *Z*, the chamber temperature *T*, and the work *W* during the mixing process can be directly obtained by setting the temperature *T*_0_, the rotor speed *N*, the processing time *t*, and the filling percentage *f*. The rate of the mechanical energy dissipation in a processing chamber $\dot{E}$ is directly related to the torque *Z* and the rotor speed *N*. When the rotor speed *N* is constant, the mechanical energy dissipation is proportional to the torque *Z*, as shown in Equation (3):

(3) $$\dot{E}=2\pi ΝΖ$$

When a certain viscous polymer melt is in a closed mixer and is in an isothermal steady-state flow, the mechanical energy dissipation can be calculated by Equation (4) [33]. Equation (5) is the formula describing the interaction of a certain viscosity of a melt with a certain geometry of the mixing cavity, where the shape of the mixing roll is approximately cylindrical and the radius is *R_i_,* *k* is the ratio of *R_i_* to the cylindrical cavity radius *R_b_*; *V_F_* is the volume of the mixing chamber; $\eta_{0}$ is the melt viscosity at low shear rates (Newtonian fluid); $\lambda_{0}^{}$ is the characteristic time of the melt, and *β* is the coefficient indicating the viscosity sensitivity of the melt to temperature. The non-Newtonian index *n* is negligible because of the temperature.

(4) $$\dot{E}=A_{n}(\kappa )fV_{F}N^{1+n}\frac{\eta_{0}}{\lambda_{0}^{1-n}}\exp\{-n\beta (T-T_{0})\}$$

(5) $$A_{n}(\kappa )=\frac{{\left(4\pi \right)}^{1+n}}{n^{n}{\left(1-\kappa^{2/n}\right)}^{n}}\times \frac{\kappa^{2}}{1-\kappa^{2}}$$

The relationship between the torque *Z*, the rotor rotational speed *N*, the non-Newtonian index *n*, and the temperature *T* can be obtained from Equations (3) and (4). Therefore, when the rotor speed *N* is constant, the shear rate γ of the material is a certain value, so the non-Newtonian index *n* of the material is also a certain value. The torque ratio of the same material at different temperatures can be obtained as [27,28,29]:

(6) $$\frac{Z_{2}}{Z_{1}}=\exp\{-n\beta (T_{2}-T_{1})\}$$

Viscosity, hence torque, depends on the temperature and MW. Variations of torque during the terminal stage of the processing could be attributed to the combined effect of melting temperature and the matrix MW change. The effect of temperature may be eliminated using a temperature-adjusted torque Z^*^:

(7) $${Ζ}^{*}=Z\times \exp\{n\beta (T-T^{*})\}$$

Where T* is an arbitrary (constant) temperature; Z* value at a constant temperature can be calculated from Equation (7). The torque value is thus only related to the MW of the polymer. The change in Z* over time given at a constant temperature indicates the change in MW caused by cross-linking, chain extension or degradation of the polymer during processing. ${\bar{Z}}^{*}$, the mean value over the selected time interval, may be taken as a measure of the rate of degradation; *R_z_* is the percentage variation of the adjusted torque per unit processing time, which is a very sensitive-albeit comparative-way to measure the rate of degradation under processing using Equation (8):

(8) $$R_{Z}=-\frac{1}{{\bar{Z}}^{*}}\frac{\Delta Z^{*}}{\Delta t}$$

The measurements were performed in an internal mixer operated at constant rotor speed; torque *Z* is directly proportional to melt viscosity *η* during the last processing stage (melt processing) [34], as expressed in Equation (9):

(9) $$Z=k_{1}\eta$$

At a given temperature and shear rate, viscosity *η* is proportional to the high power of the weight average MW of the polymer matrix, *M_w_* [35]. The non-Newtonian index *n*, viscosity *η*, *and M_w_* have the following approximate relationship:

(10) $$\eta =k_{2}M_{w}^{2.5+n}$$

where the constants *k*_1_ and *k*_2_ depend on mixer geometry, material properties, processing conditions, and temperature; *n* is the non-Newtonian index; *M_w_* is the weight average MW of the polymer. *R_M_* is used to indicate the relative rate of the change of the *M_w_* per unit processing time at a certain temperature. *R_M_* can be derived from Equations (8)–(10), approximately:

(11) $$R_{M}=-\frac{1}{{\bar{M}}_{w}}\frac{\Delta M_{w}}{\Delta t}=-\frac{1}{\Delta t}{\left(\frac{\Delta Z^{*}}{{\bar{Z}}^{*}}\right)}^{1/(2.5+n)}$$

## 3. Results and Discussion

### 3.1. Capillary Rheological Properties

**Figure 1:**
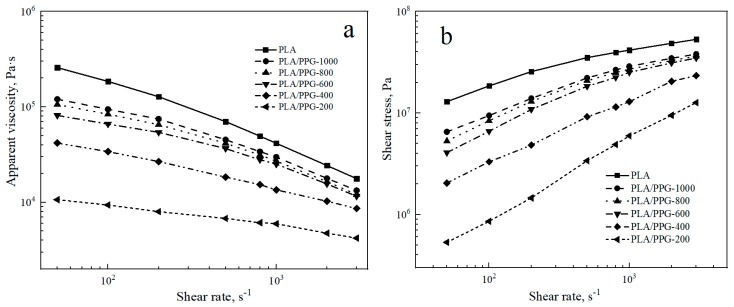
Neat polylactic acid (PLA) and polylactic acid/polypropylene glycol (PLA/PPG) composites capillary rheology test results: (**a**) Apparent viscosity versus shear rate curves, (**b**) shear stress versus shear rate curves.

The logarithmic curves of the apparent viscosity $\eta_{a}$ as a function of shear rate $\dot{\gamma }$ and the logarithmic curves of the shear stress *σ* as a function of shear rate $\dot{\gamma }$ for the PLA and PLA/PPG composites are shown in Figure 1.

Figure 1a indicates that the apparent viscosity of the PLA and PLA/PPG composites decreases with an increasing shear rate from 50 to 3000 s^−1^. The PLA and PLA/PPG composites exhibit the properties of pseudoplastic fluids. At the same shear rate, the apparent viscosity and shear stress of PLA are the highest. The apparent viscosity and shear stress of the PLA/PPG composites decrease with a decreasing PPG MW. The lower the MW of PPG, the lower the melt viscosity of the PLA/PPG composites and the better fluidity. Overall, PPG can effectively reduce the melt viscosity of PLA, especially at low MW.

The values of *n* for the PLA and PLA/PPG composites at different shear rates are listed in Table 1. As shown in Table 1, the higher the shear rate, the lower the *n* value of the PLA and PLA/PPG composites and the more pronounced non-Newtonian fluid behavior. PLA has the lowest *n* value at the same shear rate, and the *n* value of PLA/PPG-200 composite is close to a Newtonian fluid (*n* = 1) when the shear rate is lower than 100 s^−1^.

### 3.2. Torque Rheological Properties and Degradation of the PLA and PLA/PPG Composites

**Figure 2:**
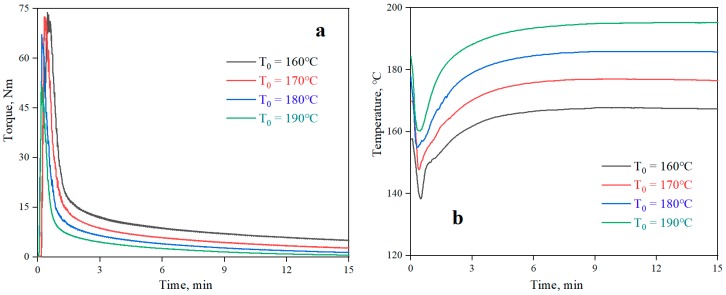
Torque (**a**) and temperature (**b**) versus time when processing the neat PLA at different chamber wall temperatures.

**Figure 3:**
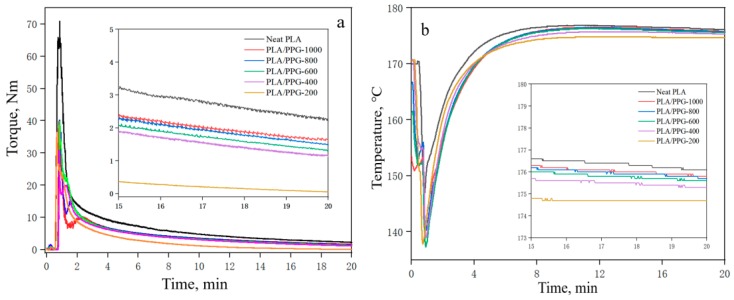
Torque (**a**) and temperature (**b**) versus time when processing the neat PLA and PLA/PPG composites.

The curves of torque and temperature versus time for the neat PLA at different processing temperatures are shown in Figure 2. The plots of torque and temperature versus time for the PLA/PPG composites mixed for 20 minutes in the torque rheometer are showed in Figure 3. From Figure 2, the torque value of the PLA at different temperatures can be obtained, and the *β* value of the PLA can be calculated from Equation (6) (0.076 °C^−1^). Meanwhile, Figure 3 reveals that the temperature of the PLA and PLA/PPG composites increased to 170 °C after 4 minutes, while the torque of the PLA and PLA/PPG composites decreased slightly, indicating that the composites were well melted under the fixed conditions. In addition, the torque values of the PLA/PPG composites were significantly lower than those of the neat PLA. The PLA/PPG composites had a lower melt viscosity and better melt flow properties, which is consistent with the capillary rheological test results.

**Figure 4:**
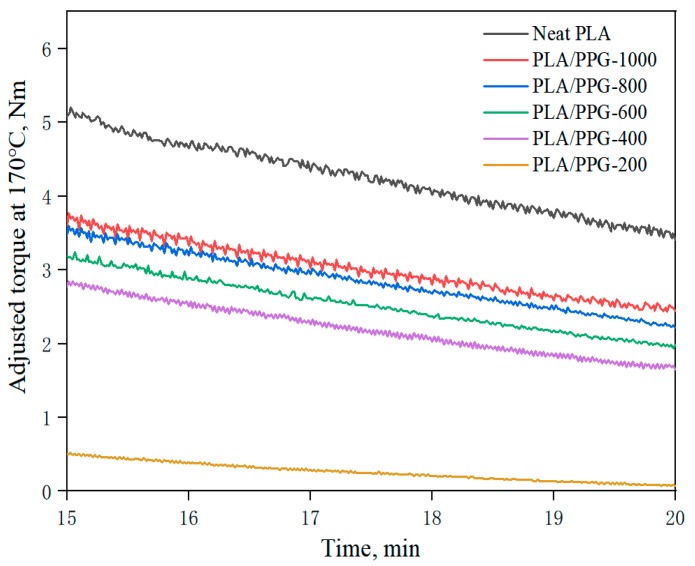
Temperature-adjusted torque versus time in the interval from 15 to 20 minutes of the neat PLA and PLA/PPG composites.

The temperature-adjusted torque is calculated using Equation (7) at a given temperature of 170 °C with the torque obtained by the torque rheological test. Figure 4 demonstrates the relationship of the temperature-adjusted torque versus time in the interval of 15 to 20 minutes of processing time of the PLA and PLA/PPG composites. Selecting the processing time of 15 to 20 minutes ensures that PLA and PPG were fully melted and uniformly mixed by that time, so the reduction of torque over time at adjusted temperature for the PLA and PLA/PPG composites was caused by degradation during processing.

**Figure 5:**
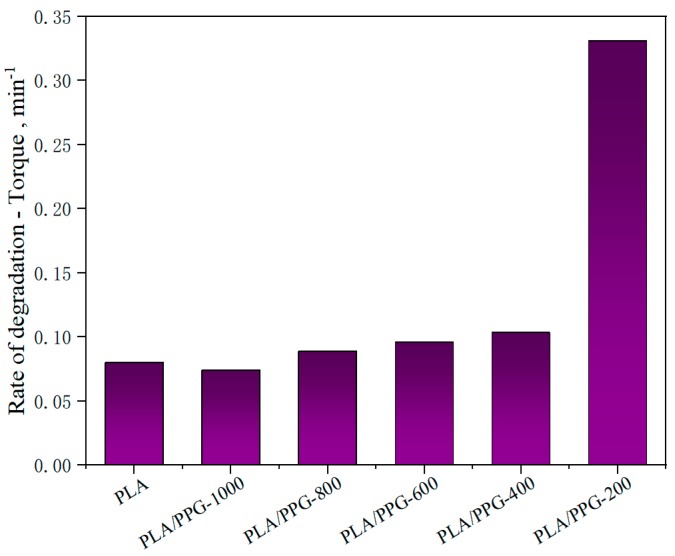
Rate of relative decrease of the temperature-adjusted torque (rate of degradation) in the interval from 15 to 20 minutes for the neat PLA and PLA/PPG composites.

**Figure 6:**
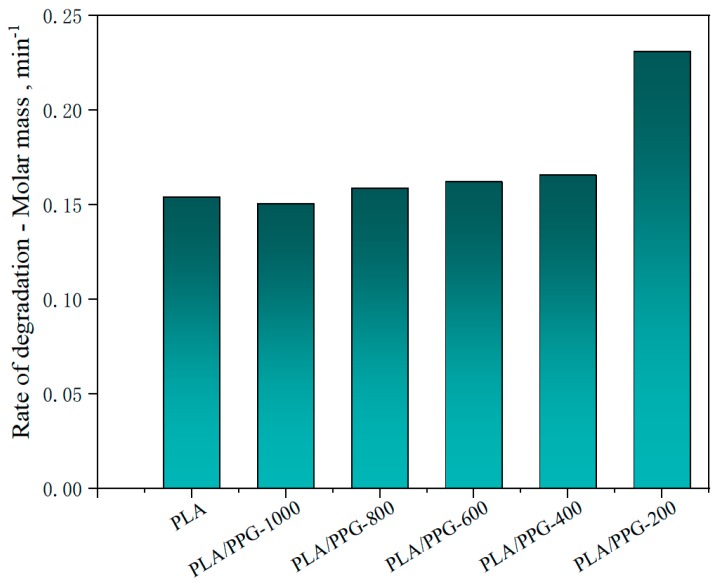
Rate of relative decrease of *M_w_* (rate of degradation) in the interval from 15 to 20 minutes for the neat PLA and PLA/PPG composites.

**Figure 7:**
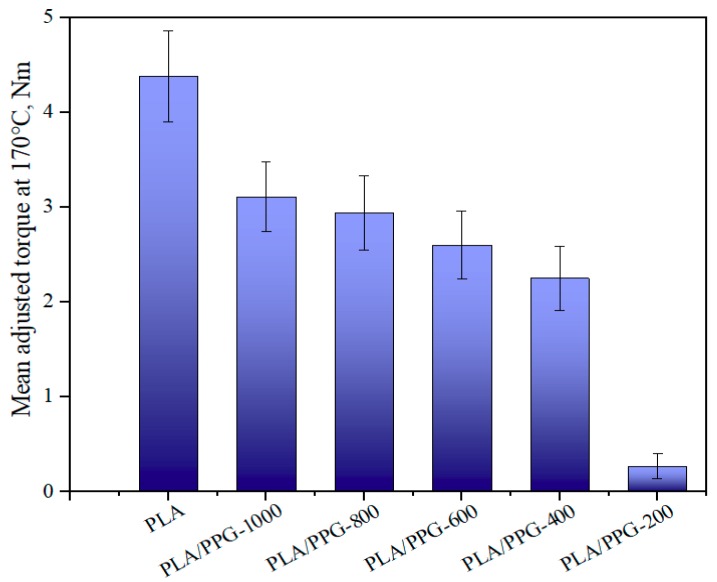
Mean value of the adjusted torque in the interval from 15 to 20 minutes for the neat PLA and PLA/PPG composites.

The corresponding data calculated by Equations (8) to (11) are presented in Figure 5, Figure 6 and Figure 7 and Table 2. The relative reduction ratio of the temperature-adjusted torque can be used to indicate the rate of degradation during processing. Figure 5 shows the rate of relative decrease of the temperature-adjusted torque (i.e., rate of degradation) in the interval from 15 to 20 minutes. As indicated in the figure, the degradation rates of PLA/PPG-800 and PLA/PPG-1000 are close to that of the neat PLA. The degradation rate increases with a decreasing PPG MW and the degradation rate of PLA/PPG-200 is the fastest.

The relative reduction ratio of the *M_w_* can also be introduced to indicate the degradation rate of the polymers during processing. Figure 6 shows the rate of relative decrease of *M_w_* (rate of degradation) in the interval from 15 to 20 minutes for the neat PLA and PLA/PPG composites, which is consistent with the trend shown in Figure 5. But the *M_w_* of the plasticized PLA after adding the PPG with different MWs does not change as significantly as the torque.

As can be seen from Equation (9), the ratio of the torque to the viscosity at the same temperature and shear rate is equal. Figure 7 presents the mean value of the adjusted torque in the interval from 15 to 20 minutes. The results indicate that the viscosity was continuously lowered when the MW of PPG was lowered. Because of the obvious degradation of PLA/PPG-200, the change of the viscosity caused by degradation is not negligible. Therefore, the torque of the materials should be higher before degradation and lower after degradation. The reduction in MW of the neat PLA is probably due to the random chain scission at the ester groups affected by both processing temperature and a trace amount of water in PLA. The addition of low MW PPG during processing introduced extra hydroxyl groups, further deteriorating the thermal stability of PLA. The transesterification reaction between PPG and PLA may result in the formation of monomers and oligomeric lactides.

### 3.3. Thermal Properties

**Figure 8:**
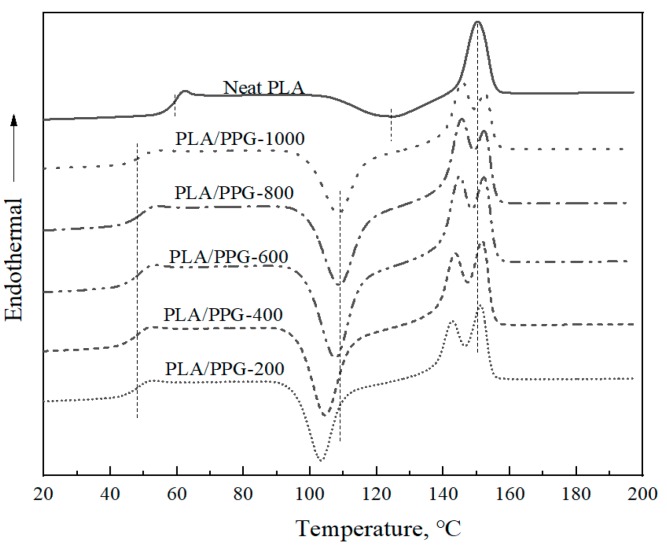
Differential scanning calorimetry (DSC) thermograms of the neat PLA and PLA/PPG composites.

The DSC thermograms of the neat PLA and PLA/PPG composites are shown in Figure 8, and the corresponding glass transition temperatures (T_g_), cold crystallization temperatures (T_cc_), and melting points (T_m_) are reported in Table 3. After being plasticized by PPG, the T_g_ of the PLA/PPG composites decreased by ca. 10 °C and two obvious melting peaks appeared. The cold crystallization temperatures of the PLA/PPG composites decreased with a decreasing PPG MW, while the glass transition temperature and melting point remained almost unchanged. Changes in T_g_, T_cc_, and T_m_ of PLA after adding PPG indicate interactions between the PLA chains and the PPG chains. 

**Figure 9:**
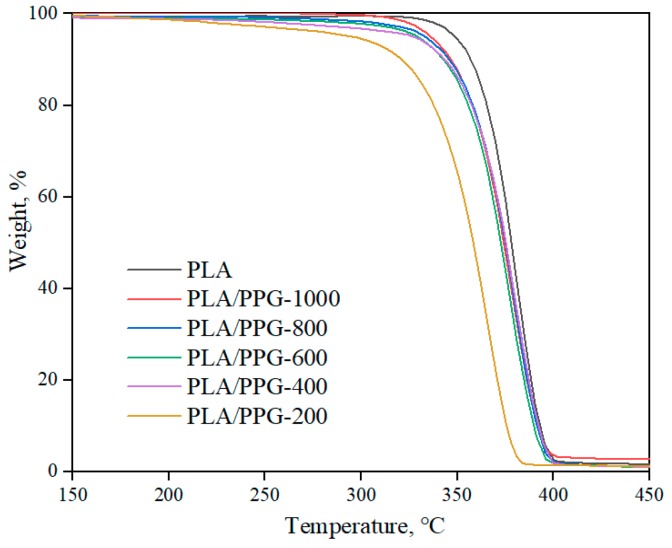
TGA thermograms of the neat PLA and PLA/PPG composites.

The TGA results (Figure 9) show that the PLA/PPG composites barely lost weight during the processing temperature range (150–180 °C), indicating that the composites at this temperature range have a good thermal stability. 

### 3.4. Micro-Morphology

**Figure 10:**
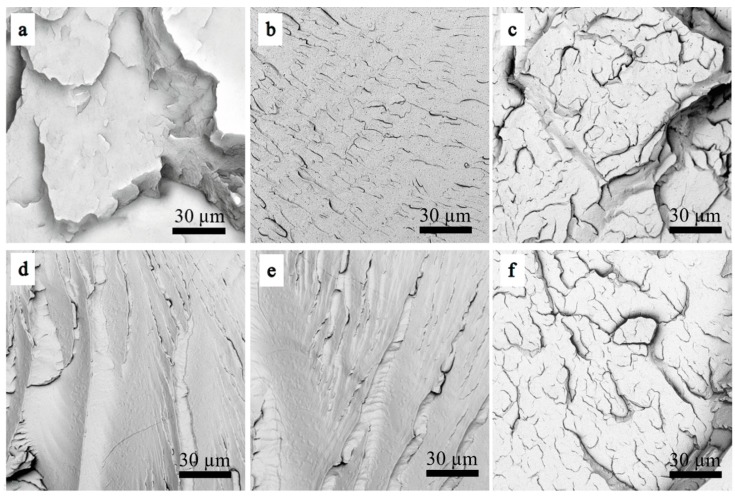
Scanning electron microscopy (SEM) images of the PLA/PPG composites: (**a**) PLA, (**b**) PLA/PPG-200, (**c**) PLA/PPG-400, (**d**) PLA/PPG-600, (**e**) PLA/PPG-800, (**f**) PLA/PPG-1000.

Figure 10 shows the micro-morphology images of the neat PLA and PLA/PPG composites. The fracture surface of the neat PLA shows an obvious brittle fracture morphology, but the PLA/PPG composites show a ductile fracture morphology. 

## 4. Conclusions

The degradation of the PLA/PPG composites containing different MW PPG during real processing was monitored by the changing trend of viscosity, which appears to be an effective and simple approach for studying the degree of degradation during PLA processing. PPG can be used to reduce the melt viscosity of PLA, and the PLA/PPG composites exhibited an obvious pseudoplastic fluid behavior. The lower the MW of PPG, the lower the melt viscosity of the resultant PLA/PPG composites. The addition of PPG leads to the degradation of PLA during processing, and the degradation of the PLA/PPG composites was more obvious when the MW of PPG was less than 400 g/mol. 

## Figures and Tables

**Table 1:** *n* values for the neat PLA and PLA/PPG composites at various shear rates.

$\dot{\gamma }$/s^−1^	*n*					
	PLA	PLA/PPG-1000	PLA/PPG-800	PLA/PPG-600	PLA/PPG-400	PLA/PPG-200
50	0.504	0.701	0.701	0.785	0.610	~1.000
100	0.428	0.596	0.596	0.667	0.518	~1.000
200	0.372	0.518	0.518	0.580	0.451	0.889
500	0.317	0.441	0.442	0.494	0.384	0.758
800	0.295	0.410	0.410	0.460	0.357	0.704
1000	0.286	0.397	0.397	0.445	0.346	0.682
2000	0.259	0.361	0.361	0.404	0.314	0.620
3000	0.246	0.343	0.343	0.384	0.298	0.588

**Table 2:** Terminal process parameters for the neat PLA and PLA/PPG composites (15–20 min).

Sample	$\bar{Z}/(N\cdot m)$	${\bar{Z}}^{*}/(N\cdot m)$	*R_Z_*/min^−1^	*R_M_*/min^−1^
PLA	2.70 ± 0.27	4.38 ± 0.48	0.0801	0.1540
PLA/PPG-1000	1.96 ± 0.22	3.11 ± 0.37	0.0739	0.1505
PLA/PPG-800	1.87 ± 0.23	2.94 ± 0.39	0.0888	0.1586
PLA/PPG-600	1.67 ± 0.22	2.60 ± 0.36	0.0959	0.1621
PLA/PPG-400	1.48 ± 0.22	2.25 ± 0.34	0.1033	0.1656
PLA/PPG-200	0.19 ± 0.09	0.27 ± 0.13	0.3311	0.2310

**Table 3:** DSC data for the neat PLA and PLA/PPG composites.

Sample	T_g_/°C	T_cc_/°C	T_m1_/°C	T_m2_/°C
PLA	58.5	124.5	150.5	-
PLA/PPG-1000	49.5	108.5	145.5	153.0
PLA/PPG-800	49.0	108.5	145.5	152.5
PLA/PPG-600	48.5	107.5	145.0	152.5
PLA/PPG-400	48.0	104.5	144.0	151.5
PLA/PPG-200	48.0	103.5	142.5	151.5
